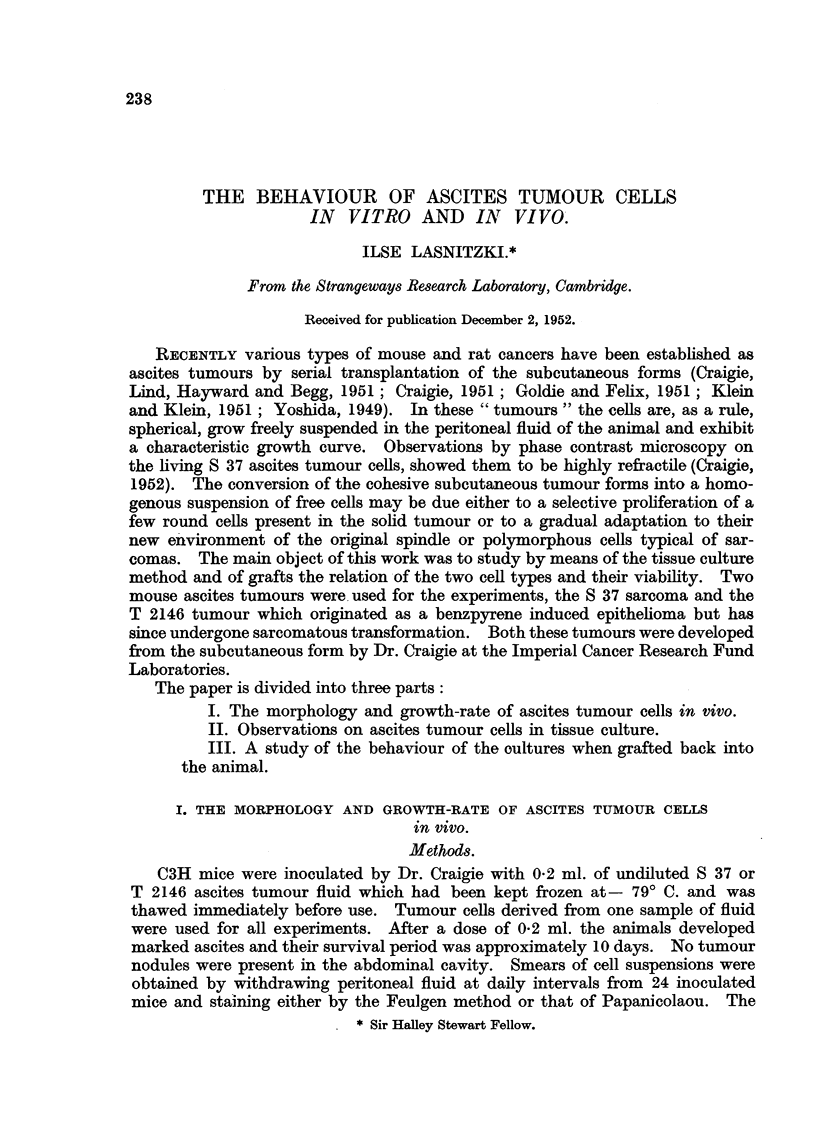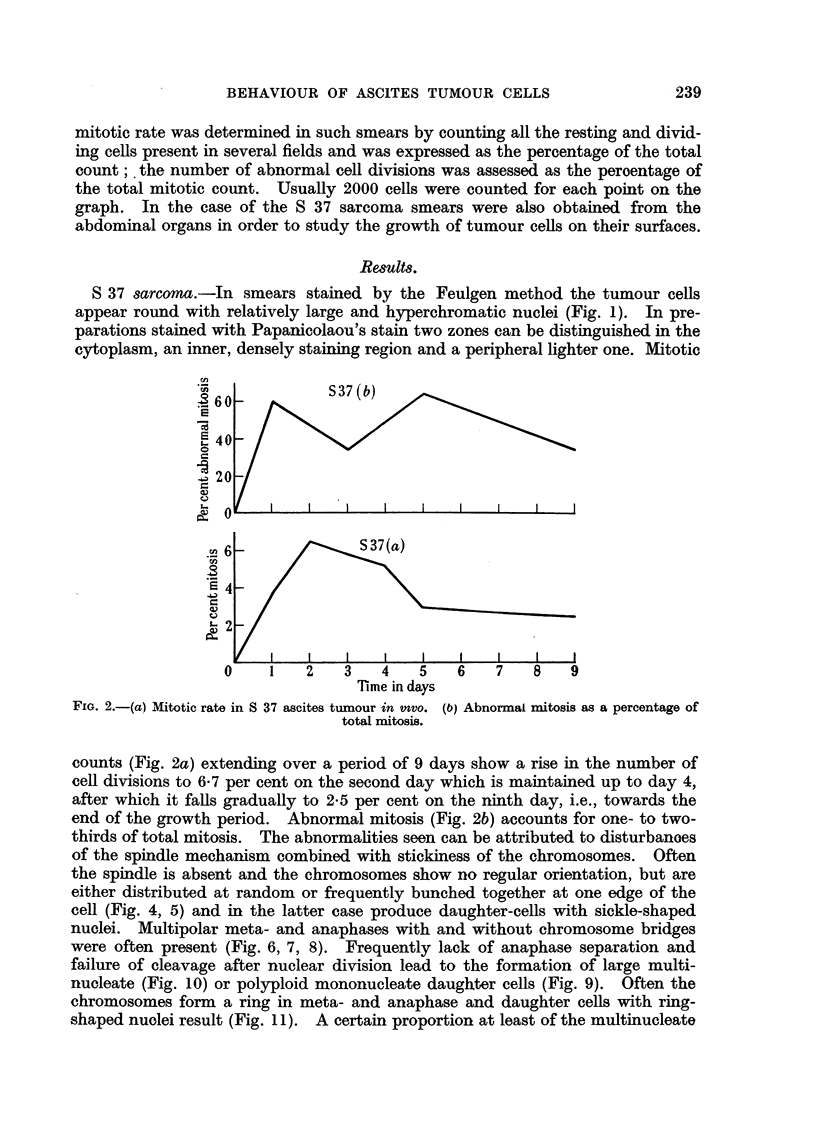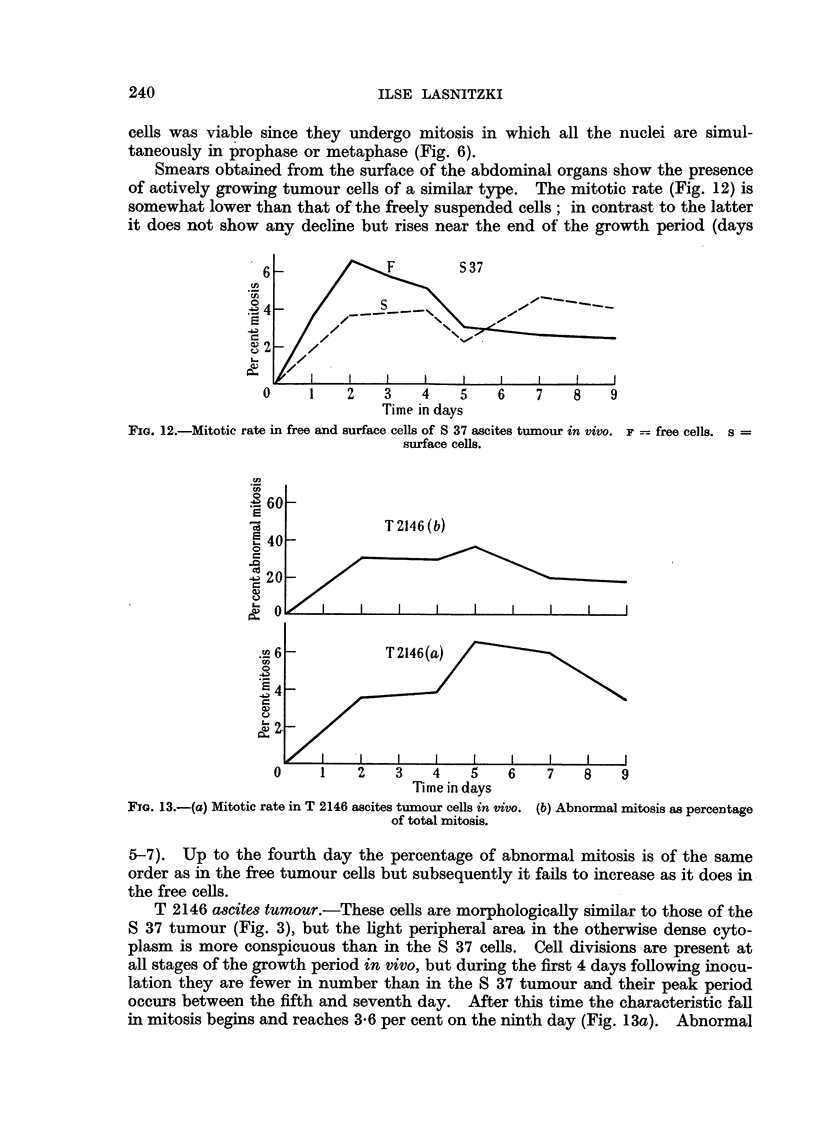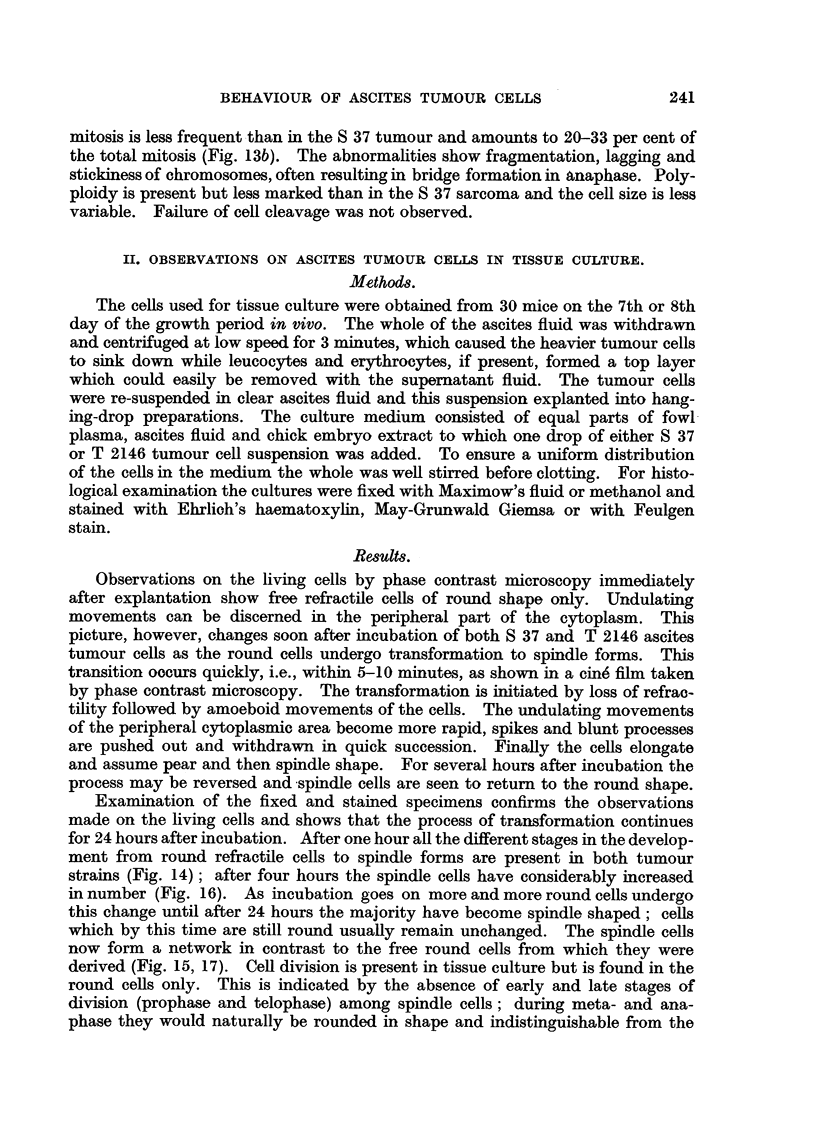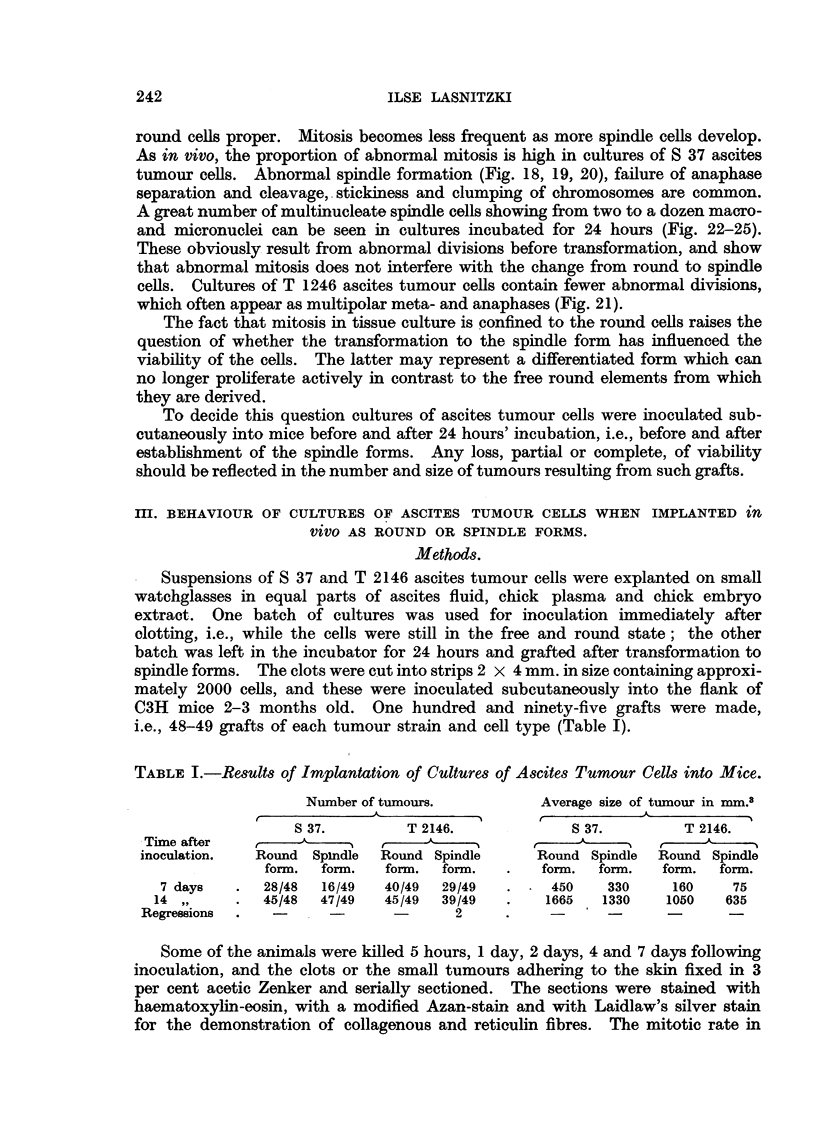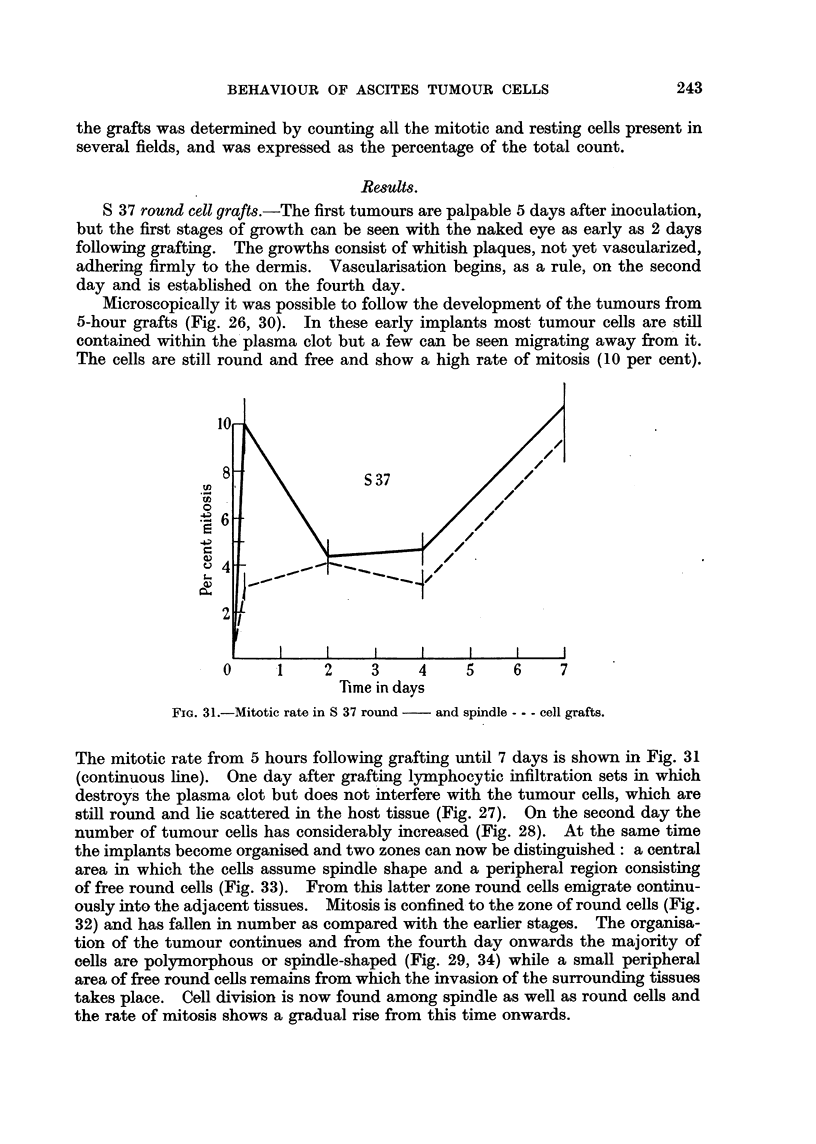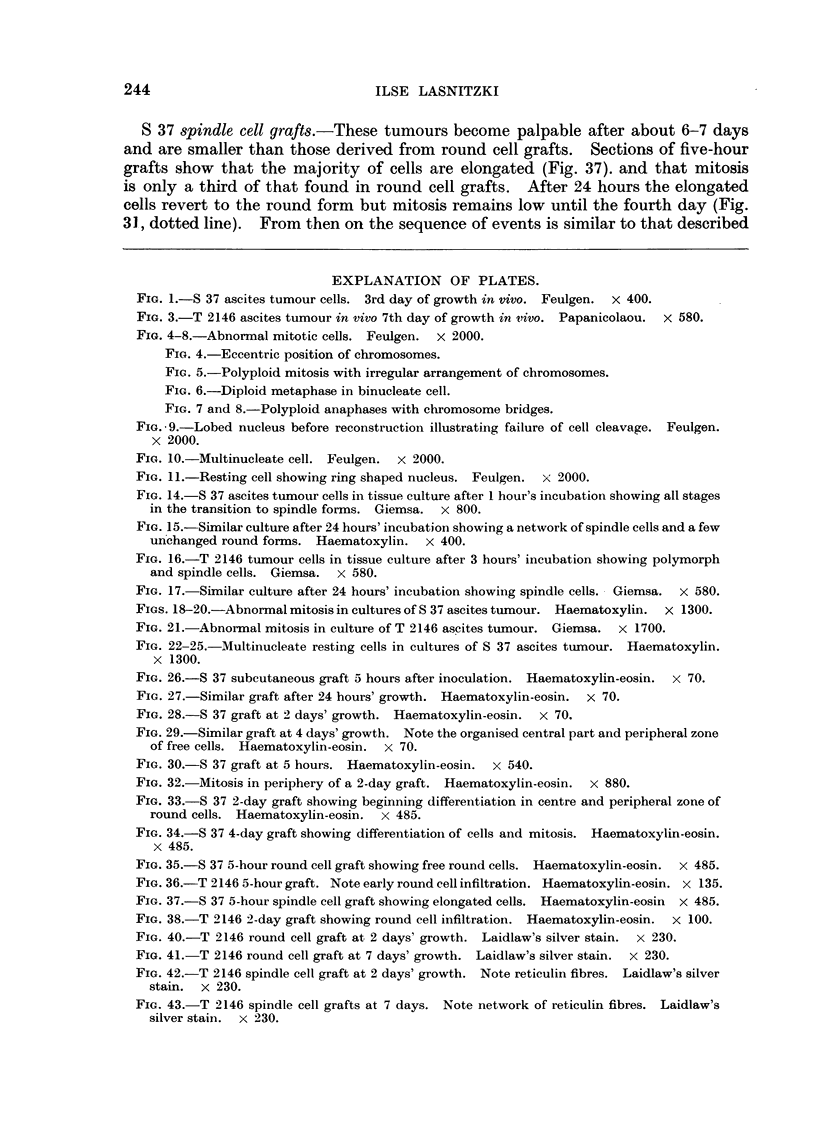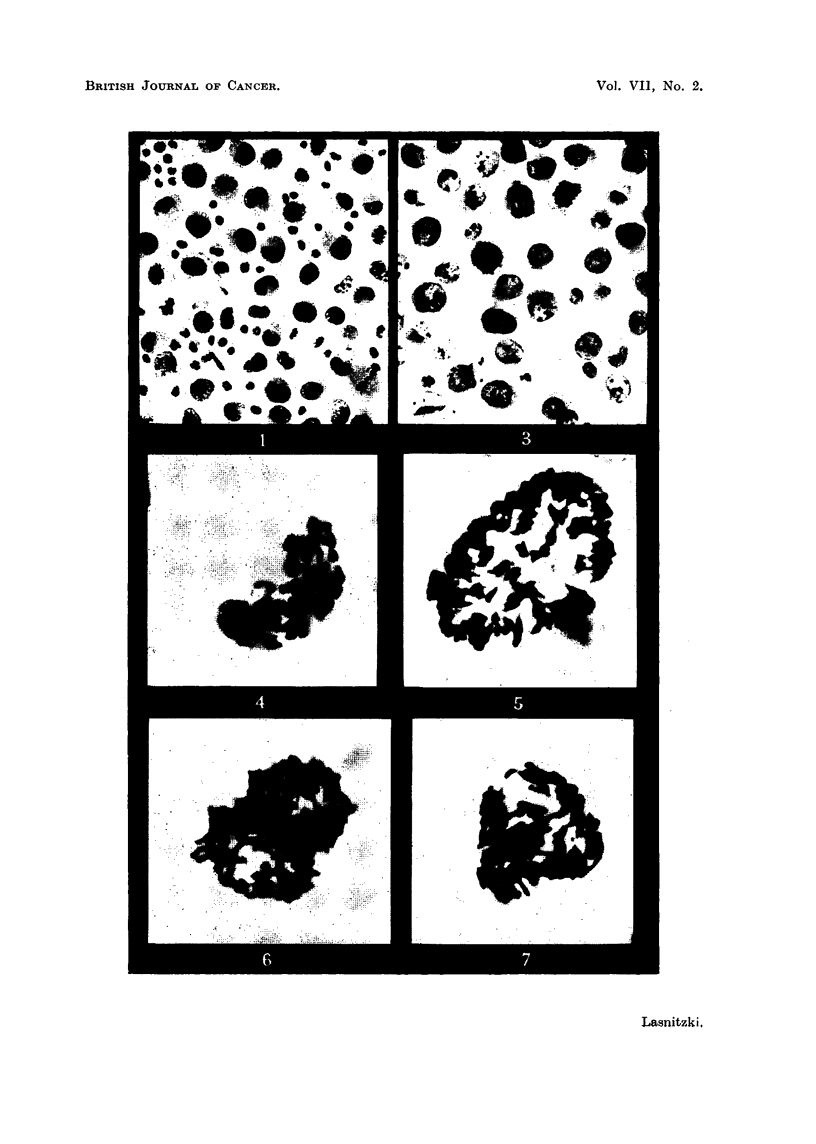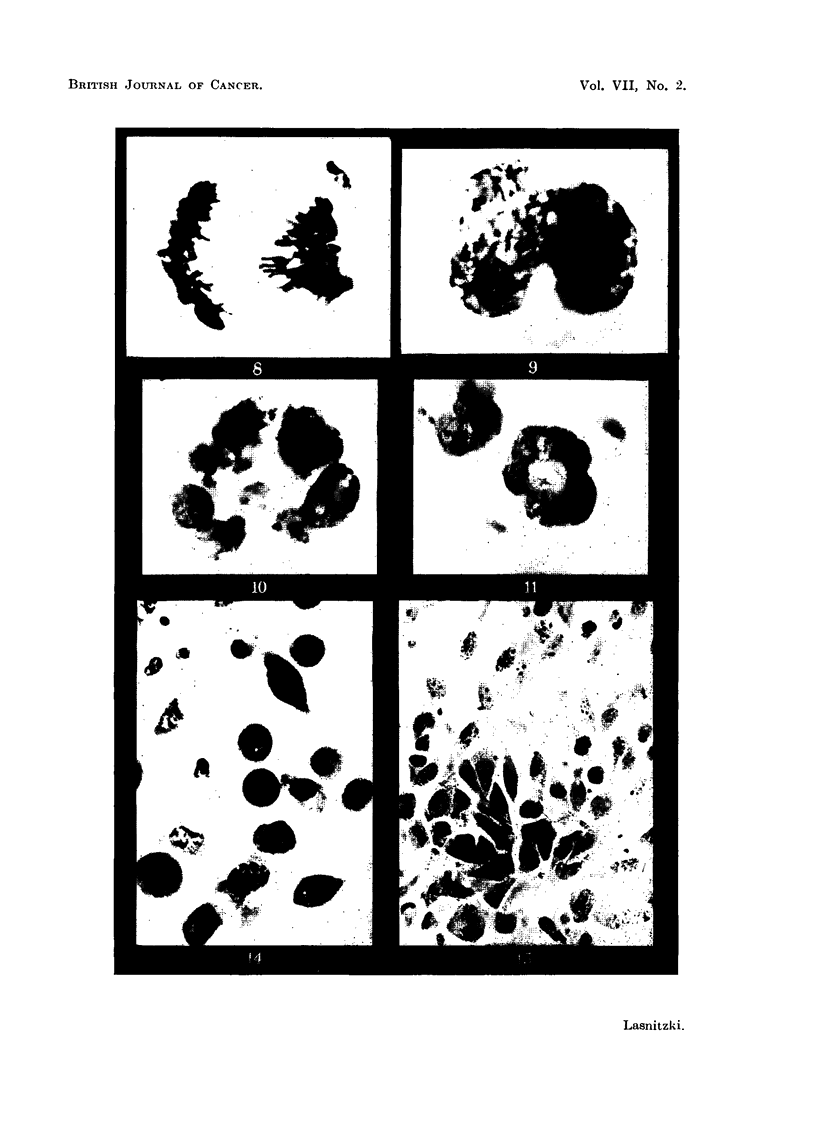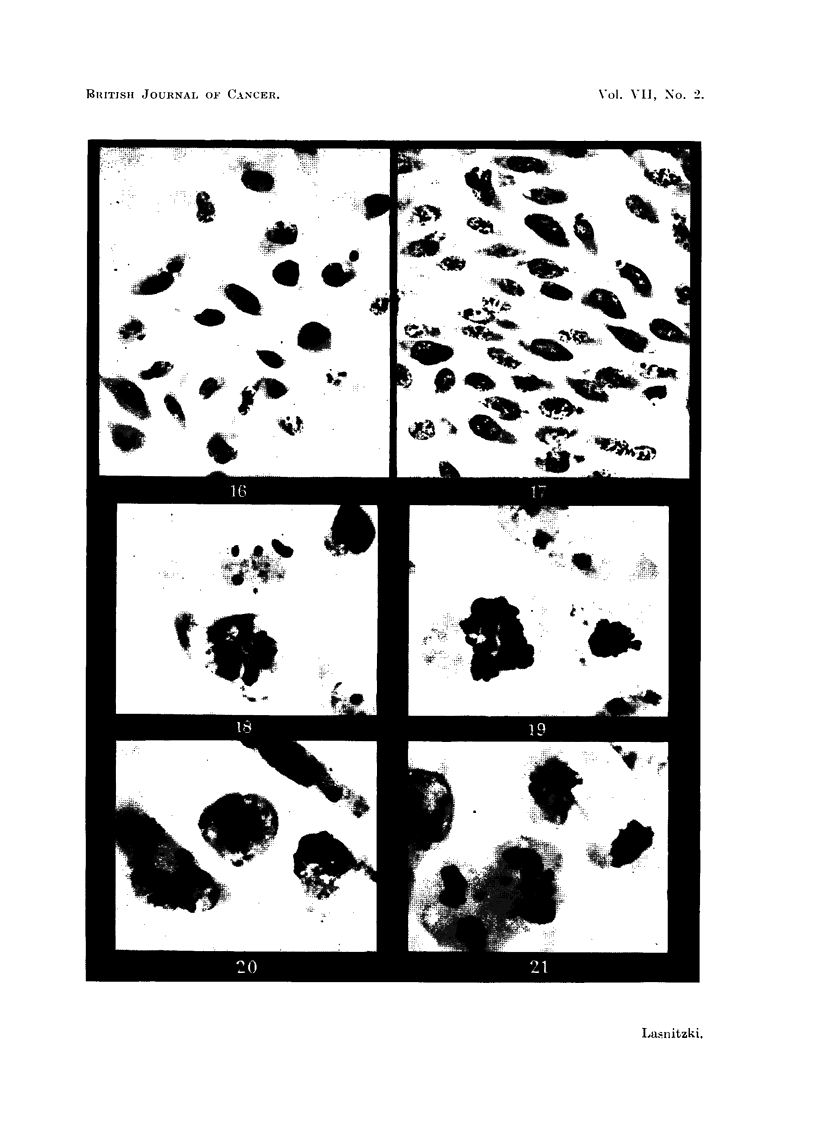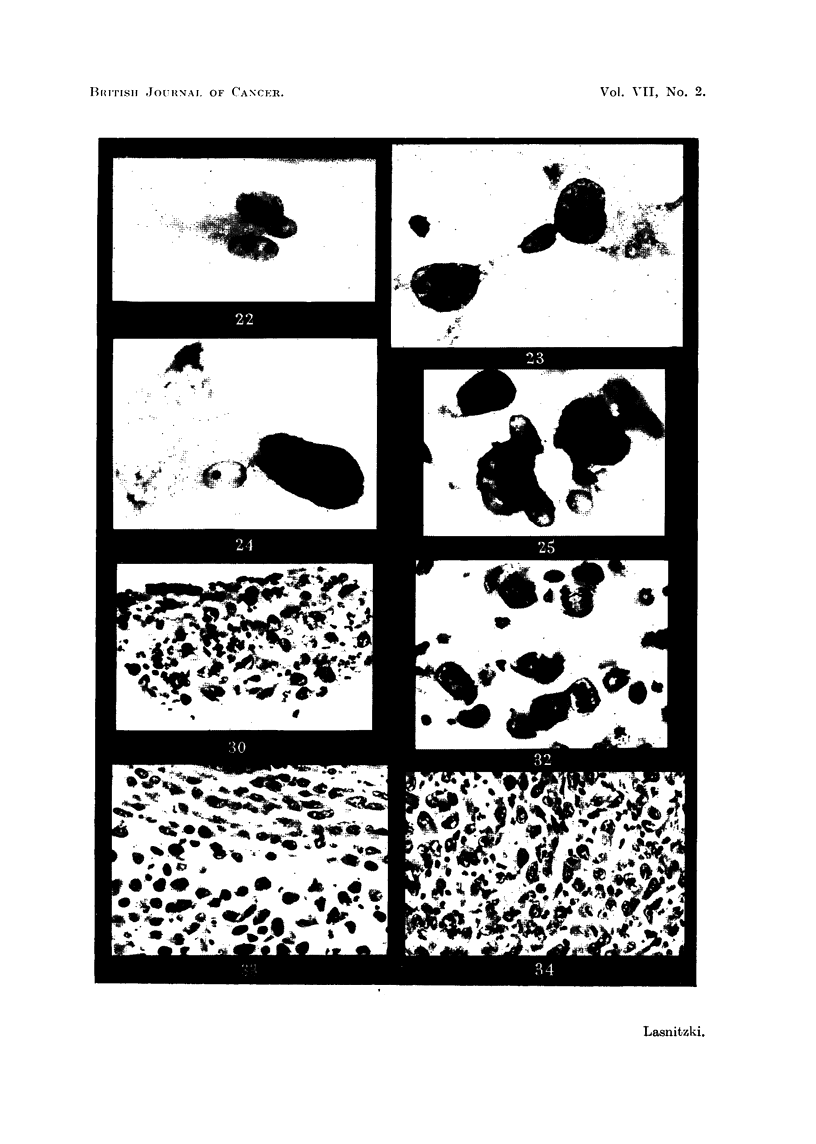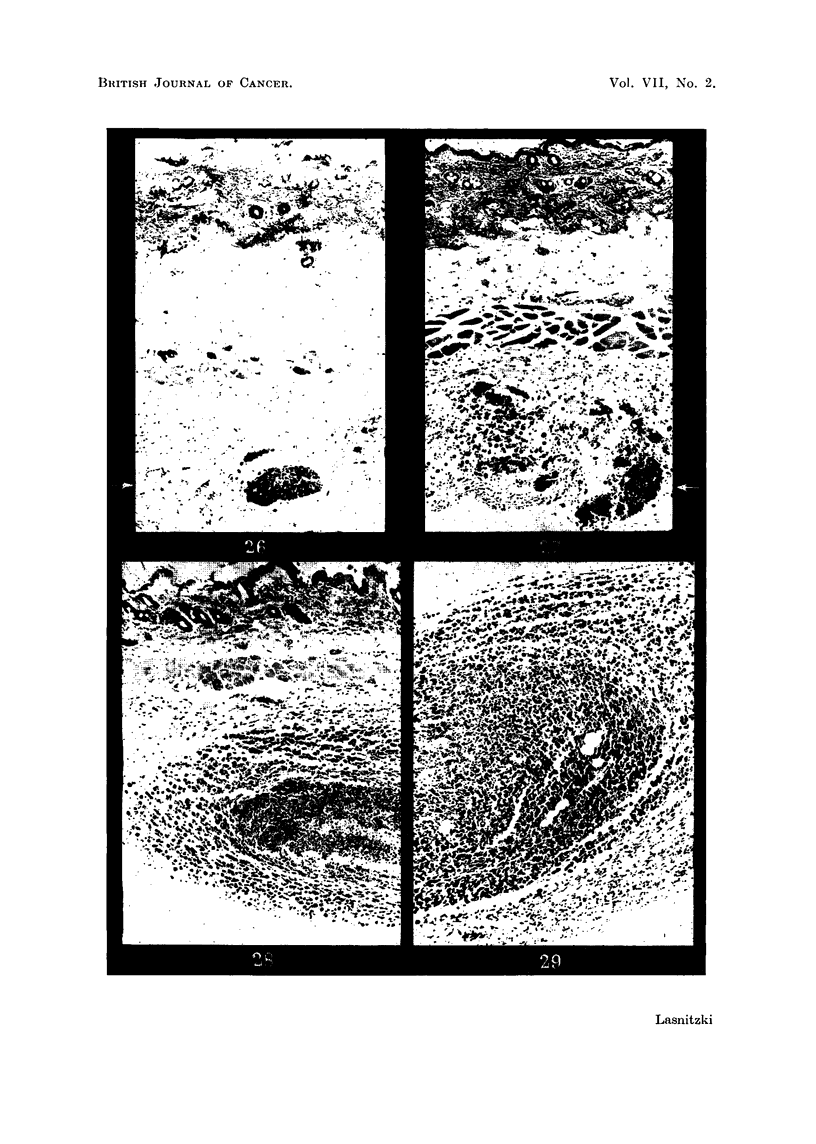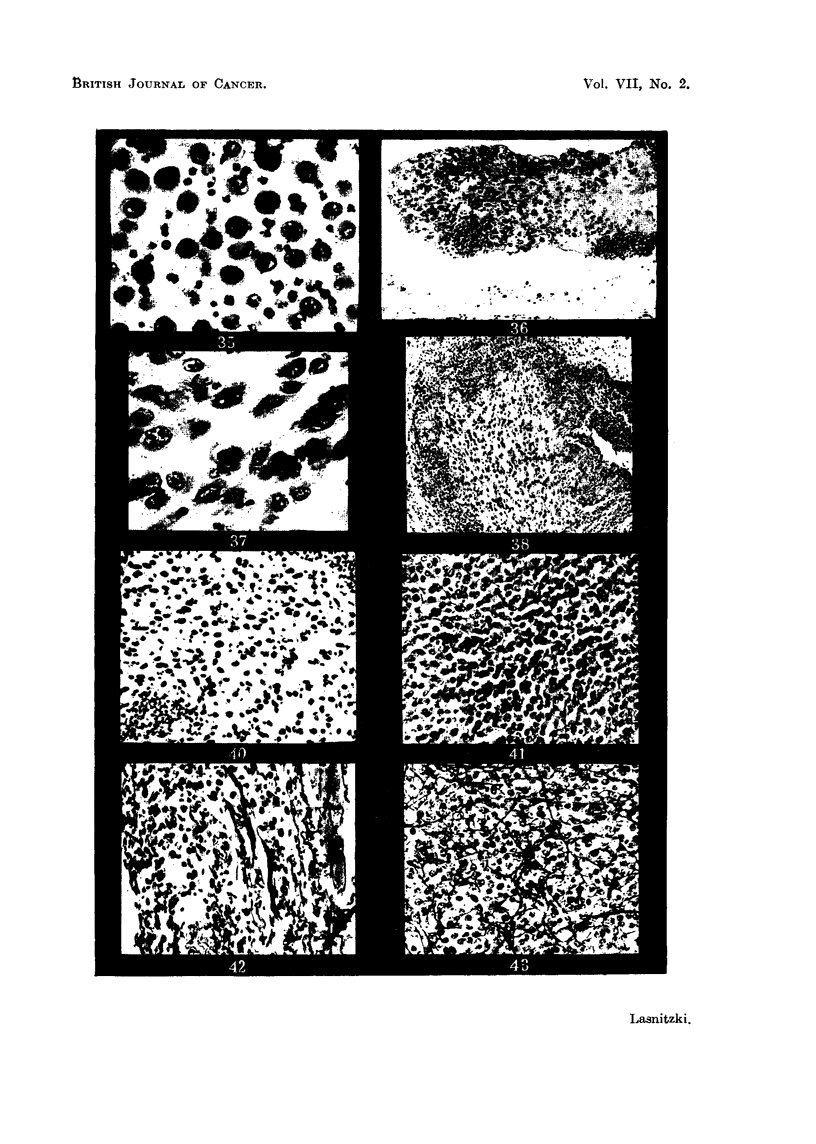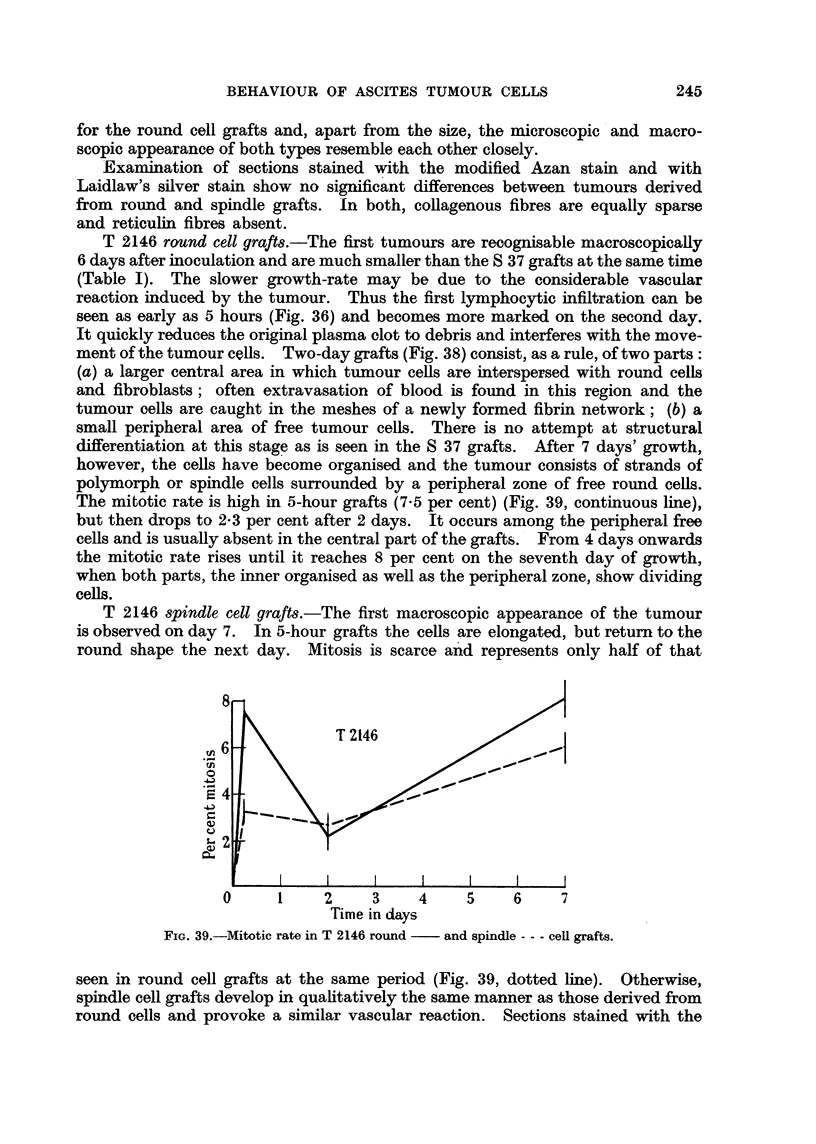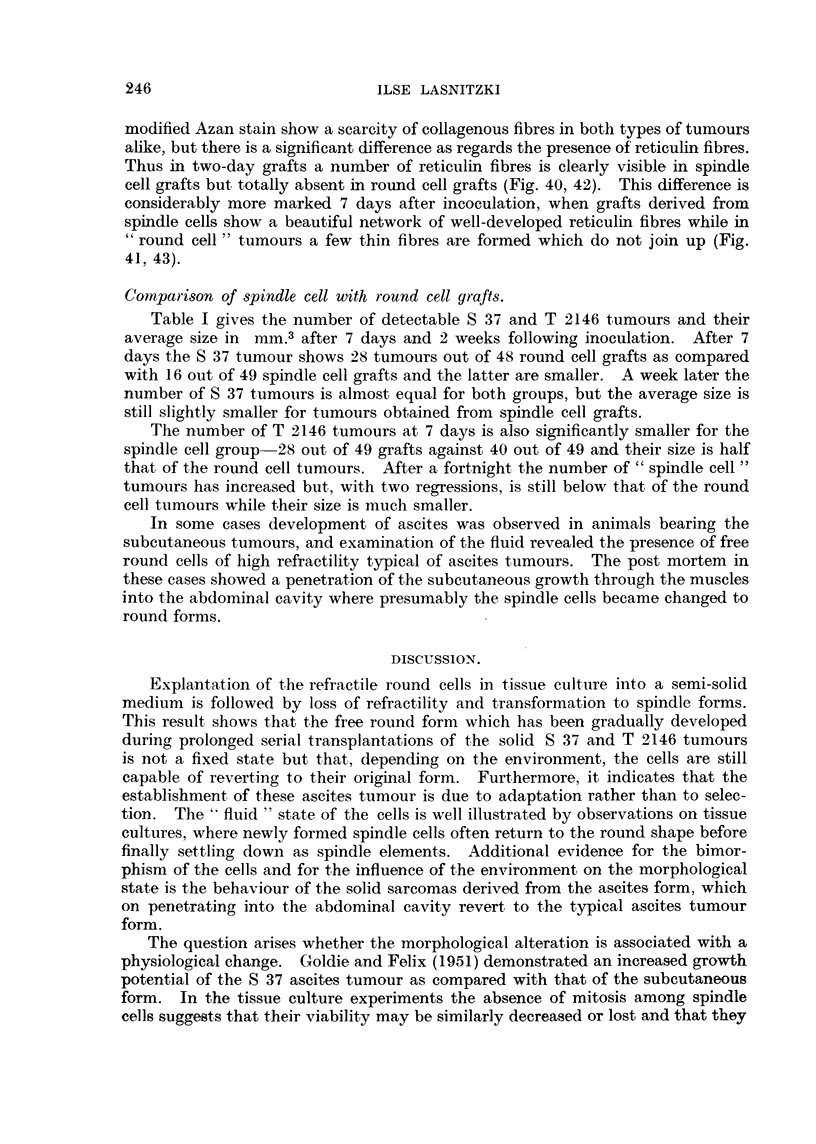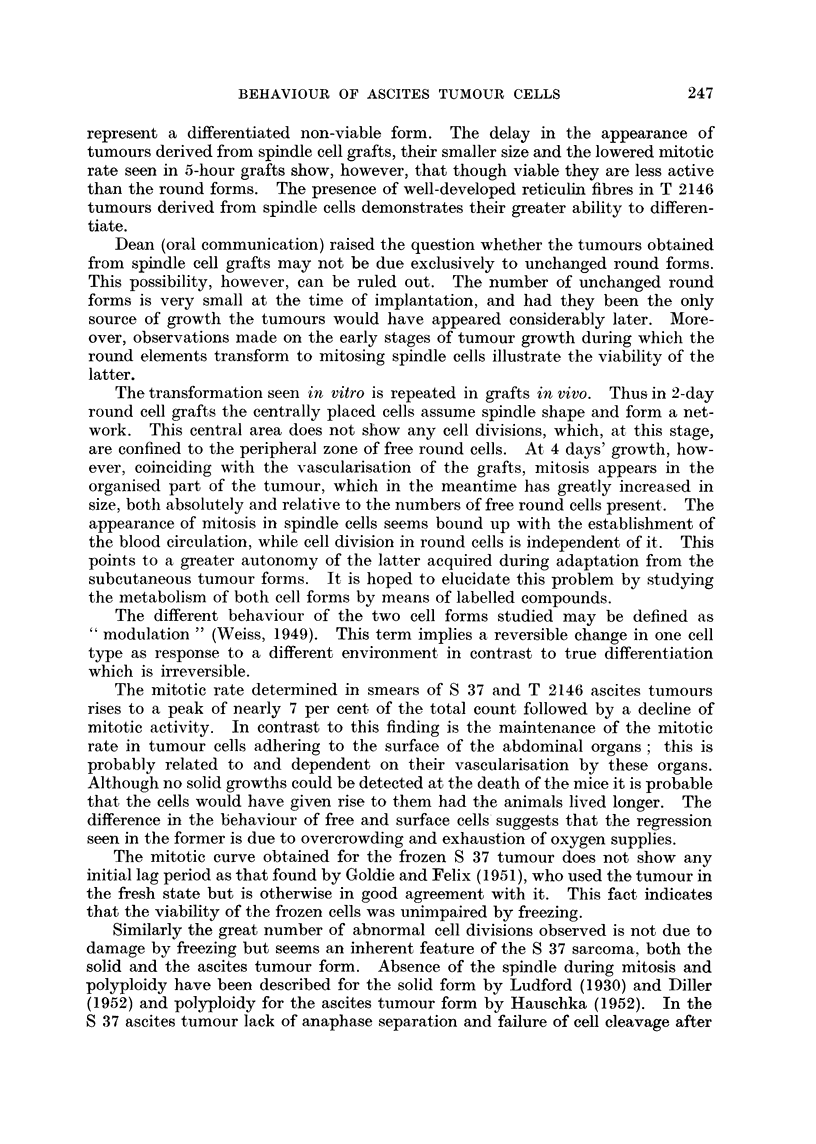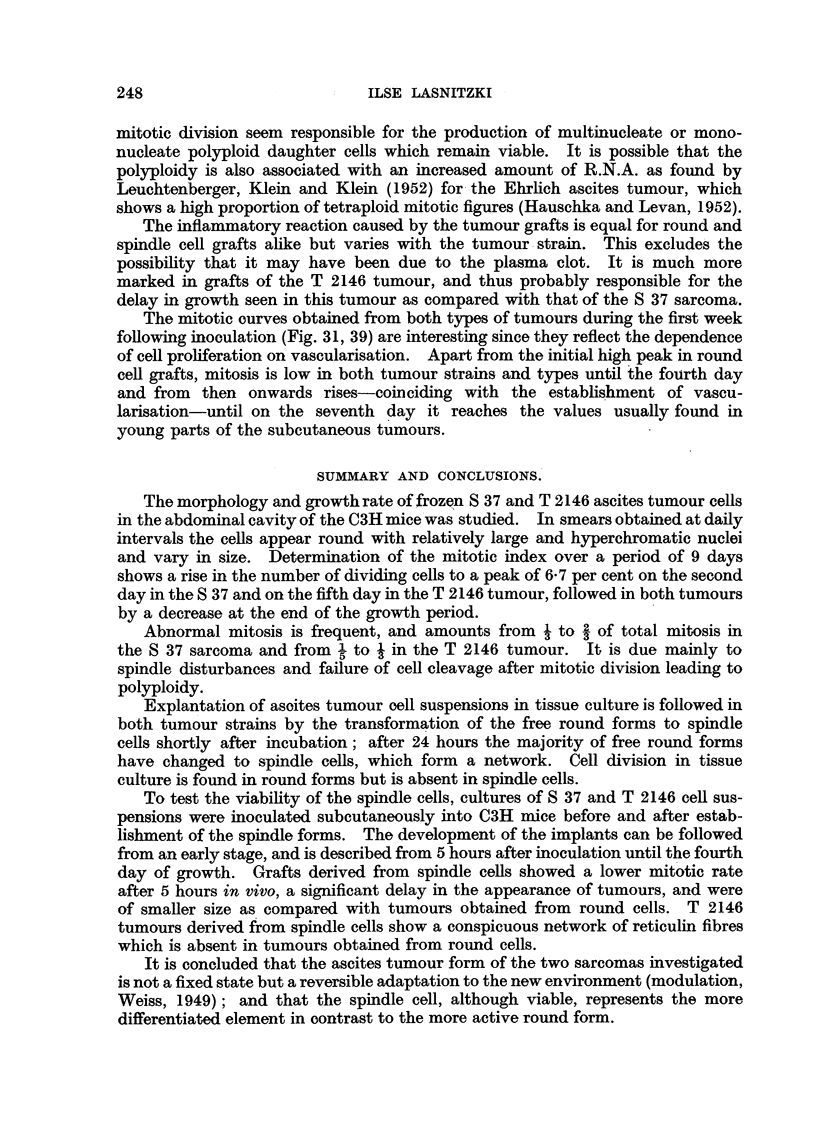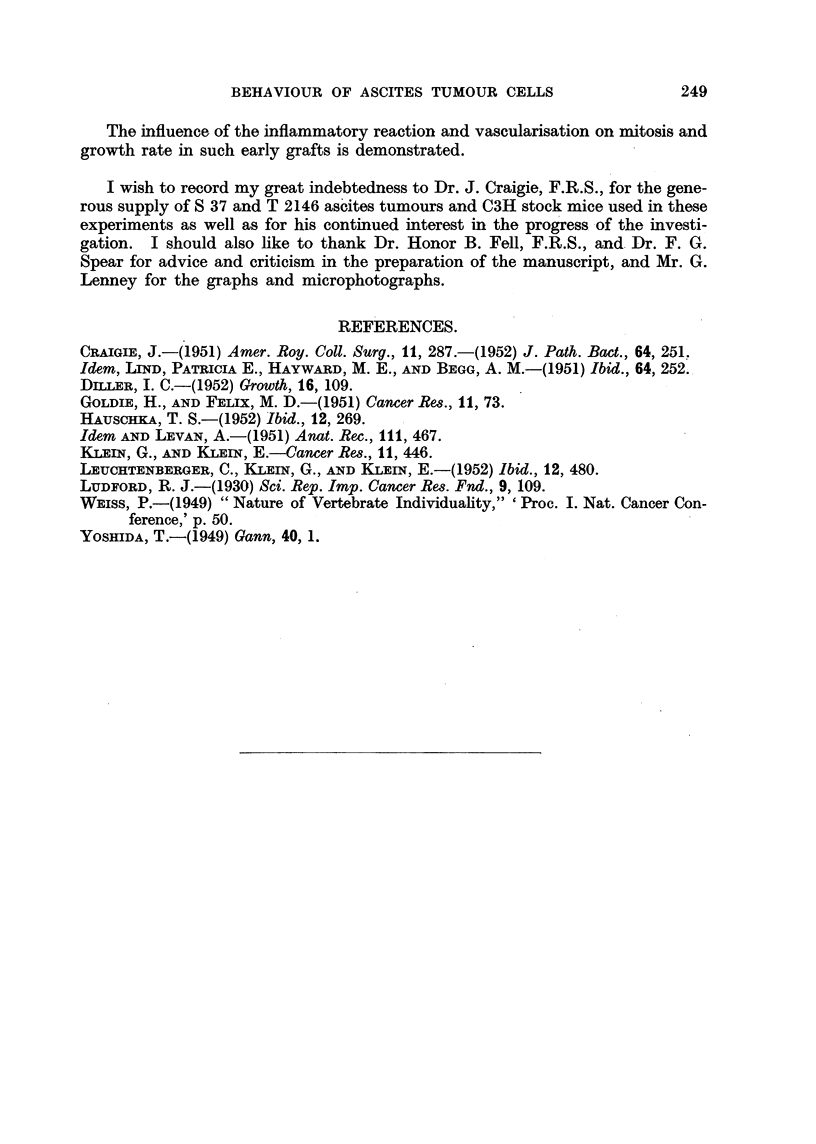# The Behaviour of Ascites Tumour Cells In Vitro and In Vivo

**DOI:** 10.1038/bjc.1953.22

**Published:** 1953-06

**Authors:** Ilse Lasnitzki

## Abstract

**Images:**


### THE BEHAVIOUR OF ASCITES TUMOUR CELLS *IN VITRO* AND *IN VIVO*.

ILSE LASNITZKI.*

*From the Strangeways Research Laboratory, Cambridge.*

Received for publication December 2, 1952.

RECENTLY various types of mouse and rat cancers have been established as ascites tumours by serial transplantation of the subcutaneous forms (Craigie, Lind, Hayward and Begg, 1951 ; Craigie, 1951 ; Goldie and Felix, 1951 ; Klein and Klein, 1951 ; Yoshida, 1949). In these " tumours " the cells are, as a rule, spherical, grow freely suspended in the peritoneal fluid of the animal and exhibit a characteristic growth curve. Observations by phase contrast microscopy on the living S 37 ascites tumour cells, showed them to be highly refractile (Craigie, 1952). The conversion of the cohesive subcutaneous tumour forms into a homogenous suspension of free cells may be due either to a selective proliferation of a few round cells present in the solid tumour or to a gradual adaptation to their new environment of the original spindle or polymorphous cells typical of sarcomas. The main object of this work was to study by means of the tissue culture method and of grafts the relation of the two cell types and their viability. Two mouse ascites tumours were used for the experiments, the S 37 sarcoma and the T 2146 tumour which originated as a benzpyrene induced epithelioma but has since undergone sarcomatous transformation. Both these tumours were developed from the subcutaneous form by Dr. Craigie at the Imperial Cancer Research Fund Laboratories.

The paper is divided into three parts :

I. The morphology and growth-rate of ascites tumour cells *in vivo*.
II. Observations on ascites tumour cells in tissue culture.
III. A study of the behaviour of the cultures when grafted back into the animal.

### I. THE MORPHOLOGY AND GROWTH-RATE OF ASCITES TUMOUR CELLS *in vivo*.

### *Methods.*

C3H mice were inoculated by Dr. Craigie with 0·2 ml. of undiluted S 37 or T 2146 ascites tumour fluid which had been kept frozen at— 79° C. and was thawed immediately before use. Tumour cells derived from one sample of fluid were used for all experiments. After a dose of 0·2 ml. the animals developed marked ascites and their survival period was approximately 10 days. No tumour nodules were present in the abdominal cavity. Smears of cell suspensions were obtained by withdrawing peritoneal fluid at daily intervals from 24 inoculated mice and staining either by the Feulgen method or that of Papanicolaou. The

* Sir Halley Stewart Fellow.

mitotic rate was determined in such smears by counting all the resting and dividing cells present in several fields and was expressed as the percentage of the total count ; the number of abnormal cell divisions was assessed as the percentage of the total mitotic count. Usually 2000 cells were counted for each point on the graph. In the case of the S 37 sarcoma smears were also obtained from the abdominal organs in order to study the growth of tumour cells on their surfaces.

*Results.*

S 37 *sarcoma.*—In smears stained by the Feulgen method the tumour cells appear round with relatively large and hyperchromatic nuclei (Fig. 1). In preparations stained with Papanicolaou's stain two zones can be distinguished in the cytoplasm, an inner, densely staining region and a peripheral lighter one. Mitotic

FIG. 2.—(*a*) Mitotic rate in S 37 ascites tumour *in vivo.* (*b*) Abnormal mitosis as a percentage of total mitosis.

counts (Fig. 2*a*) extending over a period of 9 days show a rise in the number of cell divisions to 6·7 per cent on the second day which is maintained up to day 4, after which it falls gradually to 2·5 per cent on the ninth day, i.e., towards the end of the growth period. Abnormal mitosis (Fig. 2*b*) accounts for one- to two-thirds of total mitosis. The abnormalities seen can be attributed to disturbances of the spindle mechanism combined with stickiness of the chromosomes. Often the spindle is absent and the chromosomes show no regular orientation, but are either distributed at random or frequently bunched together at one edge of the cell (Fig. 4, 5) and in the latter case produce daughter-cells with sickle-shaped nuclei. Multipolar meta- and anaphases with and without chromosome bridges were often present (Fig. 6, 7, 8). Frequently lack of anaphase separation and failure of cleavage after nuclear division lead to the formation of large multinucleate (Fig. 10) or polyploid mononucleate daughter cells (Fig. 9). Often the chromosomes form a ring in meta- and anaphase and daughter cells with ring-shaped nuclei result (Fig. 11). A certain proportion at least of the multinucleate cells was viable since they undergo mitosis in which all the nuclei are simultaneously in prophase or metaphase (Fig. 6).

Smears obtained from the surface of the abdominal organs show the presence of actively growing tumour cells of a similar type. The mitotic rate (Fig. 12) is somewhat lower than that of the freely suspended cells ; in contrast to the latter it does not show any decline but rises near the end of the growth period (days

**Fig. 12.—Mitotic rate in free and surface cells of S 37 ascites tumour *in vivo*. F = free cells. S = surface cells.**

**Fig. 13.—(*a*) Mitotic rate in T 2146 ascites tumour cells *in vivo*. (*b*) Abnormal mitosis as percentage of total mitosis.**

5–7). Up to the fourth day the percentage of abnormal mitosis is of the same order as in the free tumour cells but subsequently it fails to increase as it does in the free cells.

T 2146 *ascites tumour*.—These cells are morphologically similar to those of the S 37 tumour (Fig. 3), but the light peripheral area in the otherwise dense cytoplasm is more conspicuous than in the S 37 cells. Cell divisions are present at all stages of the growth period *in vivo*, but during the first 4 days following inoculation they are fewer in number than in the S 37 tumour and their peak period occurs between the fifth and seventh day. After this time the characteristic fall in mitosis begins and reaches 3·6 per cent on the ninth day (Fig. 13*a*). Abnormal mitosis is less frequent than in the S 37 tumour and amounts to 20–33 per cent of the total mitosis (Fig. 13*b*). The abnormalities show fragmentation, lagging and stickiness of chromosomes, often resulting in bridge formation in anaphase. Polyploidy is present but less marked than in the S 37 sarcoma and the cell size is less variable. Failure of cell cleavage was not observed.

### II. OBSERVATIONS ON ASCITES TUMOUR CELLS IN TISSUE CULTURE.

### *Methods.*

The cells used for tissue culture were obtained from 30 mice on the 7th or 8th day of the growth period *in vivo*. The whole of the ascites fluid was withdrawn and centrifuged at low speed for 3 minutes, which caused the heavier tumour cells to sink down while leucocytes and erythrocytes, if present, formed a top layer which could easily be removed with the supernatant fluid. The tumour cells were re-suspended in clear ascites fluid and this suspension explanted into hanging-drop preparations. The culture medium consisted of equal parts of fowl plasma, ascites fluid and chick embryo extract to which one drop of either S 37 or T 2146 tumour cell suspension was added. To ensure a uniform distribution of the cells in the medium the whole was well stirred before clotting. For histological examination the cultures were fixed with Maximow's fluid or methanol and stained with Ehrlich's haematoxylin, May-Grunwald Giemsa or with Feulgen stain.

### *Results.*

Observations on the living cells by phase contrast microscopy immediately after explantation show free refractile cells of round shape only. Undulating movements can be discerned in the peripheral part of the cytoplasm. This picture, however, changes soon after incubation of both S 37 and T 2146 ascites tumour cells as the round cells undergo transformation to spindle forms. This transition occurs quickly, i.e., within 5–10 minutes, as shown in a ciné film taken by phase contrast microscopy. The transformation is initiated by loss of refractility followed by amoeboid movements of the cells. The undulating movements of the peripheral cytoplasmic area become more rapid, spikes and blunt processes are pushed out and withdrawn in quick succession. Finally the cells elongate and assume pear and then spindle shape. For several hours after incubation the process may be reversed and spindle cells are seen to return to the round shape.

Examination of the fixed and stained specimens confirms the observations made on the living cells and shows that the process of transformation continues for 24 hours after incubation. After one hour all the different stages in the development from round refractile cells to spindle forms are present in both tumour strains (Fig. 14); after four hours the spindle cells have considerably increased in number (Fig. 16). As incubation goes on more and more round cells undergo this change until after 24 hours the majority have become spindle shaped; cells which by this time are still round usually remain unchanged. The spindle cells now form a network in contrast to the free round cells from which they were derived (Fig. 15, 17). Cell division is present in tissue culture but is found in the round cells only. This is indicated by the absence of early and late stages of division (prophase and telophase) among spindle cells; during meta- and anaphase they would naturally be rounded in shape and indistinguishable from the round cells proper. Mitosis becomes less frequent as more spindle cells develop. As *in vivo*, the proportion of abnormal mitosis is high in cultures of S 37 ascites tumour cells. Abnormal spindle formation (Fig. 18, 19, 20), failure of anaphase separation and cleavage, stickiness and clumping of chromosomes are common. A great number of multinucleate spindle cells showing from two to a dozen macro- and micronuclei can be seen in cultures incubated for 24 hours (Fig. 22–25). These obviously result from abnormal divisions before transformation, and show that abnormal mitosis does not interfere with the change from round to spindle cells. Cultures of T 1246 ascites tumour cells contain fewer abnormal divisions, which often appear as multipolar meta- and anaphases (Fig. 21).

The fact that mitosis in tissue culture is confined to the round cells raises the question of whether the transformation to the spindle form has influenced the viability of the cells. The latter may represent a differentiated form which can no longer proliferate actively in contrast to the free round elements from which they are derived.

To decide this question cultures of ascites tumour cells were inoculated subcutaneously into mice before and after 24 hours' incubation, i.e., before and after establishment of the spindle forms. Any loss, partial or complete, of viability should be reflected in the number and size of tumours resulting from such grafts.

### III. BEHAVIOUR OF CULTURES OF ASCITES TUMOUR CELLS WHEN IMPLANTED *in vivo* AS ROUND OR SPINDLE FORMS.

### *Methods.*

Suspensions of S 37 and T 2146 ascites tumour cells were explanted on small watchglasses in equal parts of ascites fluid, chick plasma and chick embryo extract. One batch of cultures was used for inoculation immediately after clotting, i.e., while the cells were still in the free and round state ; the other batch was left in the incubator for 24 hours and grafted after transformation to spindle forms. The clots were cut into strips 2 × 4 mm. in size containing approximately 2000 cells, and these were inoculated subcutaneously into the flank of C3H mice 2–3 months old. One hundred and ninety-five grafts were made, i.e., 48–49 grafts of each tumour strain and cell type (Table I).

TABLE I.—*Results of Implantation of Cultures of Ascites Tumour Cells into Mice.*

| Time after inoculation. | Number of tumours. | | | | Average size of tumour in mm.³ | | | |
|---|---|---|---|---|---|---|---|---|
| | S 37. | | T 2146. | | S 37. | | T 2146. | |
| | Round form. | Spindle form. | Round form. | Spindle form. | Round form. | Spindle form. | Round form. | Spindle form. |
| 7 days | 28/48 | 16/49 | 40/49 | 29/49 | 450 | 330 | 160 | 75 |
| 14 „ | 45/48 | 47/49 | 45/49 | 39/49 | 1665 | 1330 | 1050 | 635 |
| Regressions | — | — | — | 2 | — | — | — | — |

Some of the animals were killed 5 hours, 1 day, 2 days, 4 and 7 days following inoculation, and the clots or the small tumours adhering to the skin fixed in 3 per cent acetic Zenker and serially sectioned. The sections were stained with haematoxylin-eosin, with a modified Azan-stain and with Laidlaw's silver stain for the demonstration of collagenous and reticulin fibres. The mitotic rate in the grafts was determined by counting all the mitotic and resting cells present in several fields, and was expressed as the percentage of the total count.

*Results.*

S 37 *round cell grafts.*—The first tumours are palpable 5 days after inoculation, but the first stages of growth can be seen with the naked eye as early as 2 days following grafting. The growths consist of whitish plaques, not yet vascularized, adhering firmly to the dermis. Vascularisation begins, as a rule, on the second day and is established on the fourth day.

Microscopically it was possible to follow the development of the tumours from 5-hour grafts (Fig. 26, 30). In these early implants most tumour cells are still contained within the plasma clot but a few can be seen migrating away from it. The cells are still round and free and show a high rate of mitosis (10 per cent).

FIG. 31.—Mitotic rate in S 37 round —— and spindle - - - cell grafts.

The mitotic rate from 5 hours following grafting until 7 days is shown in Fig. 31 (continuous line). One day after grafting lymphocytic infiltration sets in which destroys the plasma clot but does not interfere with the tumour cells, which are still round and lie scattered in the host tissue (Fig. 27). On the second day the number of tumour cells has considerably increased (Fig. 28). At the same time the implants become organised and two zones can now be distinguished : a central area in which the cells assume spindle shape and a peripheral region consisting of free round cells (Fig. 33). From this latter zone round cells emigrate continuously into the adjacent tissues. Mitosis is confined to the zone of round cells (Fig. 32) and has fallen in number as compared with the earlier stages. The organisation of the tumour continues and from the fourth day onwards the majority of cells are polymorphous or spindle-shaped (Fig. 29, 34) while a small peripheral area of free round cells remains from which the invasion of the surrounding tissues takes place. Cell division is now found among spindle as well as round cells and the rate of mitosis shows a gradual rise from this time onwards.

S 37 *spindle cell grafts.*—These tumours become palpable after about 6–7 days and are smaller than those derived from round cell grafts. Sections of five-hour grafts show that the majority of cells are elongated (Fig. 37). and that mitosis is only a third of that found in round cell grafts. After 24 hours the elongated cells revert to the round form but mitosis remains low until the fourth day (Fig. 31, dotted line). From then on the sequence of events is similar to that described

---

EXPLANATION OF PLATES.

Fig. 1.—S 37 ascites tumour cells. 3rd day of growth *in vivo*. Feulgen. × 400.

Fig. 3.—T 2146 ascites tumour *in vivo* 7th day of growth *in vivo*. Papanicolaou. × 580.

Fig. 4–8.—Abnormal mitotic cells. Feulgen. × 2000.

Fig. 4.—Eccentric position of chromosomes.

Fig. 5.—Polyploid mitosis with irregular arrangement of chromosomes.

Fig. 6.—Diploid metaphase in binucleate cell.

Fig. 7 and 8.—Polyploid anaphases with chromosome bridges.

Fig. 9.—Lobed nucleus before reconstruction illustrating failure of cell cleavage. Feulgen. × 2000.

Fig. 10.—Multinucleate cell. Feulgen. × 2000.

Fig. 11.—Resting cell showing ring shaped nucleus. Feulgen. × 2000.

Fig. 14.—S 37 ascites tumour cells in tissue culture after 1 hour's incubation showing all stages in the transition to spindle forms. Giemsa. × 800.

Fig. 15.—Similar culture after 24 hours' incubation showing a network of spindle cells and a few unchanged round forms. Haematoxylin. × 400.

Fig. 16.—T 2146 tumour cells in tissue culture after 3 hours' incubation showing polymorph and spindle cells. Giemsa. × 580.

Fig. 17.—Similar culture after 24 hours' incubation showing spindle cells. Giemsa. × 580.

Figs. 18–20.—Abnormal mitosis in cultures of S 37 ascites tumour. Haematoxylin. × 1300.

Fig. 21.—Abnormal mitosis in culture of T 2146 ascites tumour. Giemsa. × 1700.

Fig. 22–25.—Multinucleate resting cells in cultures of S 37 ascites tumour. Haematoxylin. × 1300.

Fig. 26.—S 37 subcutaneous graft 5 hours after inoculation. Haematoxylin-eosin. × 70.

Fig. 27.—Similar graft after 24 hours' growth. Haematoxylin-eosin. × 70.

Fig. 28.—S 37 graft at 2 days' growth. Haematoxylin-eosin. × 70.

Fig. 29.—Similar graft at 4 days' growth. Note the organised central part and peripheral zone of free cells. Haematoxylin-eosin. × 70.

Fig. 30.—S 37 graft at 5 hours. Haematoxylin-eosin. × 540.

Fig. 32.—Mitosis in periphery of a 2-day graft. Haematoxylin-eosin. × 880.

Fig. 33.—S 37 2-day graft showing beginning differentiation in centre and peripheral zone of round cells. Haematoxylin-eosin. × 485.

Fig. 34.—S 37 4-day graft showing differentiation of cells and mitosis. Haematoxylin-eosin. × 485.

Fig. 35.—S 37 5-hour round cell graft showing free round cells. Haematoxylin-eosin. × 485.

Fig. 36.—T 2146 5-hour graft. Note early round cell infiltration. Haematoxylin-eosin. × 135.

Fig. 37.—S 37 5-hour spindle cell graft showing elongated cells. Haematoxylin-eosin × 485.

Fig. 38.—T 2146 2-day graft showing round cell infiltration. Haematoxylin-eosin. × 100.

Fig. 40.—T 2146 round cell graft at 2 days' growth. Laidlaw's silver stain. × 230.

Fig. 41.—T 2146 round cell graft at 7 days' growth. Laidlaw's silver stain. × 230.

Fig. 42.—T 2146 spindle cell graft at 2 days' growth. Note reticulin fibres. Laidlaw's silver stain. × 230.

Fig. 43.—T 2146 spindle cell grafts at 7 days. Note network of reticulin fibres. Laidlaw's silver stain. × 230.

Lasnitzki.

Lasnitzki.

16

17

18

19

20

21

Lasnitzki.

22

23

24

25

30

32

33

34

Lasnitzki.

26
27
28
29

35

36

37

38

40

41

42

43

Lasnitzki.

for the round cell grafts and, apart from the size, the microscopic and macroscopic appearance of both types resemble each other closely.

Examination of sections stained with the modified Azan stain and with Laidlaw's silver stain show no significant differences between tumours derived from round and spindle grafts. In both, collagenous fibres are equally sparse and reticulin fibres absent.

T 2146 *round cell grafts.*—The first tumours are recognisable macroscopically 6 days after inoculation and are much smaller than the S 37 grafts at the same time (Table I). The slower growth-rate may be due to the considerable vascular reaction induced by the tumour. Thus the first lymphocytic infiltration can be seen as early as 5 hours (Fig. 36) and becomes more marked on the second day. It quickly reduces the original plasma clot to debris and interferes with the movement of the tumour cells. Two-day grafts (Fig. 38) consist, as a rule, of two parts : (*a*) a larger central area in which tumour cells are interspersed with round cells and fibroblasts ; often extravasation of blood is found in this region and the tumour cells are caught in the meshes of a newly formed fibrin network ; (*b*) a small peripheral area of free tumour cells. There is no attempt at structural differentiation at this stage as is seen in the S 37 grafts. After 7 days' growth, however, the cells have become organised and the tumour consists of strands of polymorph or spindle cells surrounded by a peripheral zone of free round cells. The mitotic rate is high in 5-hour grafts (7·5 per cent) (Fig. 39, continuous line), but then drops to 2·3 per cent after 2 days. It occurs among the peripheral free cells and is usually absent in the central part of the grafts. From 4 days onwards the mitotic rate rises until it reaches 8 per cent on the seventh day of growth, when both parts, the inner organised as well as the peripheral zone, show dividing cells.

T 2146 *spindle cell grafts.*—The first macroscopic appearance of the tumour is observed on day 7. In 5-hour grafts the cells are elongated, but return to the round shape the next day. Mitosis is scarce and represents only half of that

FIG. 39.—Mitotic rate in T 2146 round —— and spindle - - - cell grafts.

seen in round cell grafts at the same period (Fig. 39, dotted line). Otherwise, spindle cell grafts develop in qualitatively the same manner as those derived from round cells and provoke a similar vascular reaction. Sections stained with the modified Azan stain show a scarcity of collagenous fibres in both types of tumours alike, but there is a significant difference as regards the presence of reticulin fibres. Thus in two-day grafts a number of reticulin fibres is clearly visible in spindle cell grafts but totally absent in round cell grafts (Fig. 40, 42). This difference is considerably more marked 7 days after incoculation, when grafts derived from spindle cells show a beautiful network of well-developed reticulin fibres while in " round cell " tumours a few thin fibres are formed which do not join up (Fig. 41, 43).

*Comparison of spindle cell with round cell grafts.*

Table I gives the number of detectable S 37 and T 2146 tumours and their average size in $mm.^3$ after 7 days and 2 weeks following inoculation. After 7 days the S 37 tumour shows 28 tumours out of 48 round cell grafts as compared with 16 out of 49 spindle cell grafts and the latter are smaller. A week later the number of S 37 tumours is almost equal for both groups, but the average size is still slightly smaller for tumours obtained from spindle cell grafts.

The number of T 2146 tumours at 7 days is also significantly smaller for the spindle cell group—28 out of 49 grafts against 40 out of 49 and their size is half that of the round cell tumours. After a fortnight the number of " spindle cell " tumours has increased but, with two regressions, is still below that of the round cell tumours while their size is much smaller.

In some cases development of ascites was observed in animals bearing the subcutaneous tumours, and examination of the fluid revealed the presence of free round cells of high refractility typical of ascites tumours. The post mortem in these cases showed a penetration of the subcutaneous growth through the muscles into the abdominal cavity where presumably the spindle cells became changed to round forms.

### DISCUSSION.

Explantation of the refractile round cells in tissue culture into a semi-solid medium is followed by loss of refractility and transformation to spindle forms. This result shows that the free round form which has been gradually developed during prolonged serial transplantations of the solid S 37 and T 2146 tumours is not a fixed state but that, depending on the environment, the cells are still capable of reverting to their original form. Furthermore, it indicates that the establishment of these ascites tumour is due to adaptation rather than to selection. The " fluid " state of the cells is well illustrated by observations on tissue cultures, where newly formed spindle cells often return to the round shape before finally settling down as spindle elements. Additional evidence for the bimorphism of the cells and for the influence of the environment on the morphological state is the behaviour of the solid sarcomas derived from the ascites form, which on penetrating into the abdominal cavity revert to the typical ascites tumour form.

The question arises whether the morphological alteration is associated with a physiological change. Goldie and Felix (1951) demonstrated an increased growth potential of the S 37 ascites tumour as compared with that of the subcutaneous form. In the tissue culture experiments the absence of mitosis among spindle cells suggests that their viability may be similarly decreased or lost and that they represent a differentiated non-viable form. The delay in the appearance of tumours derived from spindle cell grafts, their smaller size and the lowered mitotic rate seen in 5-hour grafts show, however, that though viable they are less active than the round forms. The presence of well-developed reticulin fibres in T 2146 tumours derived from spindle cells demonstrates their greater ability to differentiate.

Dean (oral communication) raised the question whether the tumours obtained from spindle cell grafts may not be due exclusively to unchanged round forms. This possibility, however, can be ruled out. The number of unchanged round forms is very small at the time of implantation, and had they been the only source of growth the tumours would have appeared considerably later. Moreover, observations made on the early stages of tumour growth during which the round elements transform to mitosing spindle cells illustrate the viability of the latter.

The transformation seen *in vitro* is repeated in grafts *in vivo*. Thus in 2-day round cell grafts the centrally placed cells assume spindle shape and form a network. This central area does not show any cell divisions, which, at this stage, are confined to the peripheral zone of free round cells. At 4 days' growth, however, coinciding with the vascularisation of the grafts, mitosis appears in the organised part of the tumour, which in the meantime has greatly increased in size, both absolutely and relative to the numbers of free round cells present. The appearance of mitosis in spindle cells seems bound up with the establishment of the blood circulation, while cell division in round cells is independent of it. This points to a greater autonomy of the latter acquired during adaptation from the subcutaneous tumour forms. It is hoped to elucidate this problem by studying the metabolism of both cell forms by means of labelled compounds.

The different behaviour of the two cell forms studied may be defined as " modulation " (Weiss, 1949). This term implies a reversible change in one cell type as response to a different environment in contrast to true differentiation which is irreversible.

The mitotic rate determined in smears of S 37 and T 2146 ascites tumours rises to a peak of nearly 7 per cent of the total count followed by a decline of mitotic activity. In contrast to this finding is the maintenance of the mitotic rate in tumour cells adhering to the surface of the abdominal organs ; this is probably related to and dependent on their vascularisation by these organs. Although no solid growths could be detected at the death of the mice it is probable that the cells would have given rise to them had the animals lived longer. The difference in the behaviour of free and surface cells suggests that the regression seen in the former is due to overcrowding and exhaustion of oxygen supplies.

The mitotic curve obtained for the frozen S 37 tumour does not show any initial lag period as that found by Goldie and Felix (1951), who used the tumour in the fresh state but is otherwise in good agreement with it. This fact indicates that the viability of the frozen cells was unimpaired by freezing.

Similarly the great number of abnormal cell divisions observed is not due to damage by freezing but seems an inherent feature of the S 37 sarcoma, both the solid and the ascites tumour form. Absence of the spindle during mitosis and polyploidy have been described for the solid form by Ludford (1930) and Diller (1952) and polyploidy for the ascites tumour form by Hauschka (1952). In the S 37 ascites tumour lack of anaphase separation and failure of cell cleavage after mitotic division seem responsible for the production of multinucleate or mononucleate polyploid daughter cells which remain viable. It is possible that the polyploidy is also associated with an increased amount of R.N.A. as found by Leuchtenberger, Klein and Klein (1952) for the Ehrlich ascites tumour, which shows a high proportion of tetraploid mitotic figures (Hauschka and Levan, 1952).

The inflammatory reaction caused by the tumour grafts is equal for round and spindle cell grafts alike but varies with the tumour strain. This excludes the possibility that it may have been due to the plasma clot. It is much more marked in grafts of the T 2146 tumour, and thus probably responsible for the delay in growth seen in this tumour as compared with that of the S 37 sarcoma.

The mitotic curves obtained from both types of tumours during the first week following inoculation (Fig. 31, 39) are interesting since they reflect the dependence of cell proliferation on vascularisation. Apart from the initial high peak in round cell grafts, mitosis is low in both tumour strains and types until the fourth day and from then onwards rises—coinciding with the establishment of vascularisation—until on the seventh day it reaches the values usually found in young parts of the subcutaneous tumours.

### SUMMARY AND CONCLUSIONS.

The morphology and growth rate of frozen S 37 and T 2146 ascites tumour cells in the abdominal cavity of the C3H mice was studied. In smears obtained at daily intervals the cells appear round with relatively large and hyperchromatic nuclei and vary in size. Determination of the mitotic index over a period of 9 days shows a rise in the number of dividing cells to a peak of 6·7 per cent on the second day in the S 37 and on the fifth day in the T 2146 tumour, followed in both tumours by a decrease at the end of the growth period.

Abnormal mitosis is frequent, and amounts from $\frac{1}{3}$ to $\frac{2}{3}$ of total mitosis in the S 37 sarcoma and from $\frac{1}{5}$ to $\frac{1}{3}$ in the T 2146 tumour. It is due mainly to spindle disturbances and failure of cell cleavage after mitotic division leading to polyploidy.

Explantation of ascites tumour cell suspensions in tissue culture is followed in both tumour strains by the transformation of the free round forms to spindle cells shortly after incubation; after 24 hours the majority of free round forms have changed to spindle cells, which form a network. Cell division in tissue culture is found in round forms but is absent in spindle cells.

To test the viability of the spindle cells, cultures of S 37 and T 2146 cell suspensions were inoculated subcutaneously into C3H mice before and after establishment of the spindle forms. The development of the implants can be followed from an early stage, and is described from 5 hours after inoculation until the fourth day of growth. Grafts derived from spindle cells showed a lower mitotic rate after 5 hours *in vivo*, a significant delay in the appearance of tumours, and were of smaller size as compared with tumours obtained from round cells. T 2146 tumours derived from spindle cells show a conspicuous network of reticulin fibres which is absent in tumours obtained from round cells.

It is concluded that the ascites tumour form of the two sarcomas investigated is not a fixed state but a reversible adaptation to the new environment (modulation, Weiss, 1949); and that the spindle cell, although viable, represents the more differentiated element in contrast to the more active round form.

The influence of the inflammatory reaction and vascularisation on mitosis and growth rate in such early grafts is demonstrated.

I wish to record my great indebtedness to Dr. J. Craigie, F.R.S., for the generous supply of S 37 and T 2146 ascites tumours and C3H stock mice used in these experiments as well as for his continued interest in the progress of the investigation. I should also like to thank Dr. Honor B. Fell, F.R.S., and Dr. F. G. Spear for advice and criticism in the preparation of the manuscript, and Mr. G. Lenney for the graphs and microphotographs.

### REFERENCES.

CRAIGIE, J.—(1951) *Amer. Roy. Coll. Surg.*, **11**, 287.—(1952) *J. Path. Bact.*, **64**, 251.
*Idem*, LIND, PATRICIA E., HAYWARD, M. E., AND BEGG, A. M.—(1951) *Ibid.*, **64**, 252.
DILLER, I. C.—(1952) *Growth*, **16**, 109.
GOLDIE, H., AND FELIX, M. D.—(1951) *Cancer Res.*, **11**, 73.
HAUSCHKA, T. S.—(1952) *Ibid.*, **12**, 269.
*Idem* AND LEVAN, A.—(1951) *Anat. Rec.*, **111**, 467.
KLEIN, G., AND KLEIN, E.—*Cancer Res.*, **11**, 446.
LEUCHTENBERGER, C., KLEIN, G., AND KLEIN, E.—(1952) *Ibid.*, **12**, 480.
LUDFORD, R. J.—(1930) *Sci. Rep. Imp. Cancer Res. Fnd.*, **9**, 109.
WEISS, P.—(1949) "Nature of Vertebrate Individuality," 'Proc. I. Nat. Cancer Conference,' p. 50.
YOSHIDA, T.—(1949) *Gann*, **40**, 1.